# Secure Decentralized IoT Service Platform Using Consortium Blockchain

**DOI:** 10.3390/s22218186

**Published:** 2022-10-26

**Authors:** Ruipeng Zhang, Chen Xu, Mengjun Xie

**Affiliations:** Department of Computer Science and Engineering, The University of Tennessee at Chattanooga, Chattanooga, TN 37403, USA

**Keywords:** Internet of Things, IoT communication, security and privacy, consortium blockchain, smart contract, Hyperledger fabric

## Abstract

Although many studies have been devoted to integrating blockchain into IoT device management, access control, data integrity, security, and privacy, blockchain-facilitated IoT communication is still much less studied. Blockchain has great potential in decentralizing and securing IoT communications. In this paper, we propose an innovative IoT service platform powered by the consortium blockchain technology. The proposed platform abstracts machine-to-machine (M2M) and human-to-machine (H2M) communications into services provided by IoT devices. Then, it materializes the data exchange of the IoT network through smart contracts and blockchain transactions. Additionally, we introduce the auxiliary storage layer to the proposed platform to address various off-chain data storage needs. Our proof-of-concept implementation was tested against various workloads and connection sizes under different block configurations to evaluate the platform’s transaction throughput, latency, and hardware utilization. The experimental results demonstrate that our solution can maintain high performance with a throughput of approximately 800 reads per second (RPS), 50–80 transactions per second (TPS), and a latency of 50 ms–2 s under light to moderate workloads. Our extensive evaluation of the performance impact of batch size, batch timeout, and connection size also provides valuable insights into the optimization of blockchain configuration for achieving high performance.

## 1. Introduction

Integration of the IoT and blockchain began to bloom thanks to distributed ledger technologies and cryptocurrencies. Previous IoT systems depend on centralized servers for communication and data storage, which often become the single point of risk to the security and privacy of the systems. Blockchain technologies, however, enable collaboration between untrusted parties in a decentralized manner. They eliminate the need for a trusted intermediary by creating a self-organized transaction network guided by a consensus protocol. Thanks to the distributed network architecture, data on blockchains not only have high availability but also strong integrity assured by cryptography algorithms and the immutable data structure. As a result, blockchain technologies are widely applied to fields of IoT, such as supply chains [1], power grids [2], healthcare [3], and smart homes [4].

However, prominent blockchain solutions have their performance, security, and privacy concerns. First, public permissionless blockchains, such as Bitcoin and Ethereum, oblige no restrictions on the participants who can create blocks and read transactions [5]. In the context of IoT, the openness of such blockchains endangers user privacy and exposes IoT systems to cyber attacks. In addition, the anonymity of permissionless blockchains makes it challenging to audit operations and trace in the network. Second, to reach global state consistency in a trustless environment, public permissionless blockchains use costly consensus protocols, such as proof of work (PoW), proof of stake (PoS), or protocols that require particular hardware (e.g., proof of elapsed time) [6]. Such protocols are often not suitable for IoT systems where devices are heterogeneous and power-constrained. It is also difficult for them to meet the throughput and latency requirements of IoT applications, which often demand hundreds of transactions to be committed to the ledger within milliseconds to seconds.

Consortium blockchains, in comparison, remedy those disadvantages of public permissionless blockchains in a semi-trusted environment. Unlike permissionless blockchains, a consortium blockchain is operated by a group of collaborating entities, and only authorized nodes of these entities can commit blocks to the ledger [7]. On a consortium blockchain, the ledger can also be made visible to all members or part of the group via access control policies. Since participating authorities manage blockchain actors, it is more feasible to trace transaction flows and construct audit trails. Regarding consensus protocols, consortium blockchains assume that transaction validators are predefined and semi-trusted. Less resource-demanding consensus protocols, such as practical byzantine fault tolerance (PBFT) [8] and raft [9] can be utilized in consortium blockchains to improve scalability. Therefore, consortium blockchains usually yield much better performance than public permissionless blockchains [10].

### 1.1. Motivation

We reviewed existing IoT and blockchain integration in literature and discovered a research gap in realizing decentralized secure and scalable M2M and H2M communication with consortium blockchain technology. That is, overcoming the downsides of public permissionless blockchains for IoT communications by replacing them with consortium blockchains. A few research groups [11,12,13] explored the application of permissioned consortium blockchains and smart contracts in securing IoT communications and sensor data. However, they are either highly focused on a specific blockchain application of IoT, lack a robust system design, or miss an extensive evaluation of their proposed approach. There is a strong need for a more generalized, clearly defined, and extensively evaluated framework for IoT communications utilizing the consortium blockchain technology.

### 1.2. Contribution

In this paper, we present a decentralized IoT service platform, called DISP, for secure M2M and M2H communications inside an IoT environment based on a permissioned consortium blockchain. Instead of implementing a specific application of IoT with blockchain, the proposed work aims to establish a generic communication framework for various IoT systems. The consortium blockchain is a secure and scalable communication channel for IoT devices and applications in our solution. The communication protocol is formulated as services defined by IoT devices and provided to applications. Meanwhile, exchanging messages become blockchain transactions and are conveyed through the blockchain network. In addition, optional auxiliary storage is introduced to fit the proposed framework into diverse application scenarios, such as sensor data archives and real-time messaging. Finally, the framework provides a lightweight software development kit (SDK) and platform gateways to simplify blockchain operations for resource-constrained IoT devices and application developers. Compared to related studies, the proposed framework DISP has the following advantages:

(1) *Generality and versatility*: DISP is designed to power a wide range of IoT applications. It abstracts the communication protocol into the services that IoT devices can customize. It also imposes minimum assumptions about the underlying blockchain features or storage types. For example, it does not rely on a specific function offered by a particular blockchain platform or use a dedicated storage solution exclusively. Therefore, it can support various IoT applications to satisfy communication and data processing requirements.

(2) *Interoperability*: DISP can be integrated into existing IoT systems smoothly. With the help of straightforward SDKs and platform gateways, an application developer can not only bring new IoT devices to the proposed framework but also migrate legacy IoT devices or systems easily. Meanwhile, DISP works well with existing IoT identity management and access control infrastructure and can reuse digital identities already in place.

(3) *High performance*: As illustrated by the performance evaluation results, our framework has considerable read and write throughputs and reasonable latencies even under high workloads. DISP works much more efficiently for semi-trusted consortium environments than permissionless blockchain-based IoT communication solutions, which rely on heavy consensus protocols.

Our contributions to this work are as follows:

(1) We propose a novel consortium blockchain-based IoT platform, namely DISP, for secure and decentralized IoT communications. DISP models IoT communications as services powered by smart contracts and blockchain transactions. In this paper, we elaborate on the system design and implementation that make DISP capable of supporting diverse IoT applications.

(2) We evaluate DISP extensively to demonstrate its performance under various workloads and block configurations. The experimental results illustrate that DISP can achieve high read and write throughputs and reasonable latencies for most workloads. This work also discusses how DISP can address security and privacy concerns.

(3) Two use-case studies, Parrot and Crystal Ball, are presented to showcase the versatility of DISP. Additionally, the DISP project—including its SDKs, use case demos, and testbed set-up scripts—is an open-source project to promote the reproducibility of this research.

### 1.3. Paper Organization

The remainder of this paper is organized as follows: Section 2 reviews related work of blockchain-based IoT identity management and access control, data storage and marketplace, and device manipulation. Section 3 describes the proposed IoT service platform’s architecture and key processes. Section 4 briefly presents the proof of concept platform implementation and several use-case studies. Then, we evaluate the performance of the proposed platform with a series of experiments and discuss its security and privacy implications in Section 5. Finally, Section 6 concludes this paper with future research directions.

## 2. Related Work

The integration of blockchain and IoT has been extensively studied since the emergence of blockchain technology. Attempts have been made to address the challenges of IoT, such as distributed and heterogeneous architecture, device, data security, and profitability utilizing blockchains [14]. In this section, we will review the current development of IoT blockchain integration in three specific areas related to our proposed service platform: identity management and access control, data storage and marketplace, and device command and control.

### 2.1. Identity Management and Access Control

Identity management (IdM) and access control are keys to security and trust of IoT devices and data. It is difficult to apply traditional IdM systems to IoT environments, especially distributed and collaborative ones, due to their centralized nature, security vulnerabilities, and service fragmentation [15]. IoT’s security, scalability, and interoperability requirements call for new IdM and access control paradigms, and blockchain-based solutions are promising answers.

One approach to building a blockchain-based IdM is recreating public key infrastructure (PKI) using blockchain and smart contracts [16,17,18,19]. A blockchain-based PKI supports the same critical operations, such as registration, verification, and revocation as a traditional centralized PKI, with improvements in security and privacy. Other research takes a different approach by building identity systems tailored to specific blockchain implementation. For instance, Sovrin [20] and its underlying Hyperledger Indy blockchain [21] provide a full-stack solution to decentralized, self-sovereign IdM on a public permissioned blockchain. Finally, storing identities off-chain (e.g., in traditional PKI) and linking them back to the blockchain were also discussed and utilized in projects, such as Hyperledger Fabric [22].

Once identity management has been established for an IoT system, one can further introduce access control to IoT data and regulate the communications between devices. Traditional access control methods, including role-based access control (RBAC), attribute-based access control (ABAC), and capability-based access control (CBAC) are less capable of supporting the enormous, heterogeneous, and decentralized IoT environments. Considering how access decisions are made, existing blockchain-based access control methods can be categorized into (1) transaction-based access control and (2) smart contract-based access control [23]. Transaction-based access control methods such as FairAccess [24] leverage blockchain as an immutable, distributed storage for access tokens, while the generation and verification of those tokens take place off-chain. On the other hand, smart contract-based access control focuses more on decentralizing the decision-making process with smart contracts. For example, IoTChain [25] allows resource owners to define smart contracts for granting client access and generating access tokens. IoT-CCAC [26] presents a consortium blockchain-based CBAC approach for IoT applications that exchange data between different consortium members.

### 2.2. Data Storage and Marketplace

The rapid advancement in distributed ledger technology invites new opportunities for distributed data storage, data sharing, and data monetization. Blockchain is a distributed, immutable database system where IoT data, such as sensor readings and access logs, may be stored. However, due to the block size limitation and scalability considerations, storing IoT data off-chain is more practical. Thus, hybrid blockchain-based storage networks have been proposed to reduce the chances of a single point of failure (SPOF) as seen in traditional centralized storage systems, provide data integrity and security, and lower the storage cost [27].

For instance, general-purpose blockchain-based solutions that support bulk data storage, such as Storj [28], Sia [29], and FileCoin [30], store only metadata of data blocks on the blockchain to prevent the bloating issue. On such platforms, files are split into smaller blocks and sent to the underlying distributed storage network composed of miners or storage nodes. To encourage participation and prevent dishonest behavior and free-riding, they also introduce new consensus protocols and cryptocurrencies to compensate for miners’ storage and bandwidth usage.

The rise of blockchain technology and IoT also accelerates the growth of the market of IoT data. With blockchain technology, an IoT data marketplace can become fully decentralized and autonomous, while reducing cost, improving transaction efficiency, and promoting data privacy. Research in this area has been centered around ensuring data authenticity and provenance, secure data transfer, and payment processing [31,32,33,34]. Additionally, industry-led initiatives such as IOTA [35], XBR [36], and Streamr [37] also provide real-world insights into the monetization of IoT data on the blockchain.

### 2.3. Device Manipulation

Blockchain technology also enables fully decentralized M2M and H2M communications for IoT systems. With smart contracts, a blockchain-based IoT system is capable of autonomous decision-making based on business logic [14]. Such systems are relieved from centralized device management and control and, hence, less vulnerable to a SPOF. Meanwhile, the blockchain and smart contract ensure communication integrity and provide an immutable audit trail.

Slock.it [38] is among the first applications that leverage blockchain and smart contracts for controlling embedded devices. It envisages a blockchain-based economy of things where people can rent their unused assets, such as bikes, to others through smart contracts. A user can discover and lease assets on the Slock.it platform, and the whole process is enabled by smart asset controllers or IoT devices connected to the Ethereum network. Upon successful payment, an asset will be unlocked by its controller as instructed by smart contracts.

Apart from securely sharing physical assets, Ethereum has been used as a decentralized M2M channel for IoT, thanks to its popularity and versatility. Fakhri and Mutijarsa [39] proposed a proof-of-concept demonstration on replacing MQTT [40] with the Ethereum blockchain for communications between IoT devices. The devices can talk to each other by reading data from and writing data to the blockchain via a smart contract intermediary. Wickström et al. [41] introduced an Ethereum-based protocol for IoT device management and task handling. This protocol utilizes two smart contracts to register an IoT device and create tasks for it. The authors pointed out that their protocol could reduce network attack risks to IoT devices because they can use the blockchain as a secure channel for remote command and control while disallowing other incoming network connections.

Finally, several studies have implemented decentralized IoT device management and communication using permissioned blockchain framework. Compared to permissionless blockchains, a permissioned blockchain inherently integrates with identity management and authentication so that the participation of the consensus can be verified and authorized. It usually comes with better scalability and energy efficiency because its consensus can be achieved without computationally expensive mining. Ali et al. [11] discussed a Hyperledger fabric-based IoT architecture for a smart home scenario where every device stores and shares its data via blockchain transactions. In such an environment, a smart device can request services from other devices by communicating directly or indirectly through the cloud. A smart contract also guards the list of devices and their shared secret keys. Hang and Kim [12] outlined a blockchain platform for securing IoT sensing data integrity. The platform provides smart contracts for registering and querying IoT devices and creating and deploying tasks that IoT devices can process. In the end, a device owner can receive notifications about the events generated by the tasks from the blockchain. The authors also detailed the implementation of their proposed platform that utilizes Hyperledger Fabric. Zhang et al. [13] applied Hyperledger Fabric to facilitate secure device communications in the edge computing environment. However, device communications and manipulations on their proposed platform are implemented through tasks that are scheduled and executed outside the blockchain.

### 2.4. Comparison of DISP and Related Work

A comparison of the proposed platform DISP with the aforementioned related studies is presented in Table 1. The research domain, research goal, applied blockchain technology, access control availability, and data storage mechanism are considered in the comparison. Distinct from other research, DISP focuses on securing the communication between IoT devices and their users. To realize this goal, we introduce authentication, access control, and data storage capabilities to DISP. DISP can be integrated into a broad spectrum of IoT scenarios rather than just a specific area of IoT. Moreover, DISP is the only one that explicitly considers the multi-organizational environment among all the compared studies. DISP also outperforms most related work in terms of system throughput and latency. The detailed performance comparison is given in Section 5.

## 3. System Design

**Figure 1:**
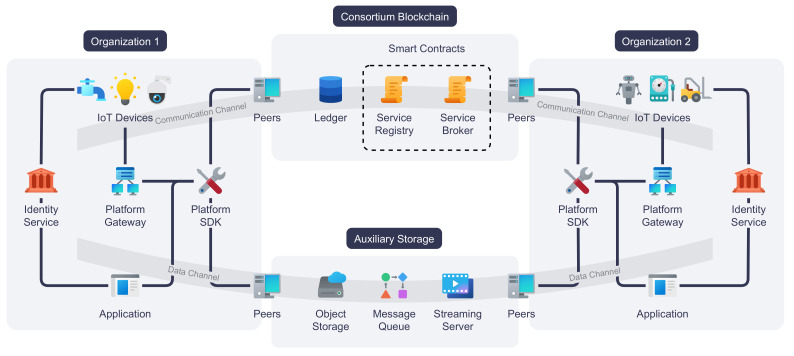
Consortium blockchain-based IoT service platform architecture.

An architectural overview of our proposed IoT service platform i.e., DISP, is illustrated in Figure 1. This overview shows the platform paradigm for a consortium composed of two organizations for demonstration purposes. At its core, the platform is powered by a consortium blockchain. Peer nodes from two organizations together serve the distributed ledger and smart contracts. These nodes also execute smart contracts when requested by the blockchain actors, which can be IoT devices or applications, and endorse blockchain transactions. IoT devices from any organization within the consortium may connect to the platform directly or through the platform gateway that handles blockchain operations on the device’s behalf. To facilitate the integration of various IoT devices and accelerate application development, we also incorporate an SDK to reduce the complexity of devices and applications to interact with the platform. At least one identity service is required to provide digital identities to each IoT device, application, and peer node in every organization of the consortium, although multiple organizations may share the same identity service. Finally, auxiliary storage is introduced to enable data sharing between different organizations. The actual storage types and implementations are affected by individual application requirements.

The following are the core components of DISP:

(1) *IoT devices*: IoT devices connect the physical space to cyberspace. Regarding DISP, they are the primary service providers. Services provided by IoT devices either measure or affect their environment, given that the device is a sensor or actuator. For example, a thermometer service reports the temperature of its surroundings, while a thermostat provides the service of adjusting room temperature. An IoT device may connect to the blockchain directly or through a platform gateway if its computational resources are constrained.

(2) *Platform gateway*: A platform gateway bridges the communication between IoT devices and the platform. Often, an IoT device is not able to participate in blockchain transactions directly due to energy or computational power constraints or because it is not programmable. In this case, it can delegate blockchain operations to the platform gateway without changing its inherent communication protocols.

(3) *Consortium blockchain*: At the core of the proposed platform, a consortium blockchain is employed as the distributed ledger that records all IoT devices and their services. It is also the primary communication channel between IoT services and their consumers, offering better performance and scalability compared to permission-less public blockchains. The IoT network can become decentralized, meaning there are no longer centralized servers that are often SPOF. Moreover, the platform can leverage access control features introduced by consortium blockchains to secure IoT services from unauthorized access. Finally, since all changes to the IoT services and the service request and responses are recorded immutably on the ledger, the blockchain can essentially serve as a data historian for data auditing.

(4) *Peer*: Peers, or peer nodes, are computers that perform blockchain operations or offer auxiliary storage to IoT devices and applications of the platform. When serving as blockchain peers, they are responsible for hosting the distributed ledger and executing smart contracts. They are also essential in transaction endorsement. As for the auxiliary storage, a peer can be tailored to specific application needs. For instance, it can be a distributed network storage node, a distributed message queue broker, or a proxy server of media streams. Any organization in the consortium can contribute peers to the network. It is crucial to ensure a fair amount of peers from different organizations to achieve meaningful decentralization, and peers should be placed close to their neighboring IoT devices and gateways to reduce network overhead.

(5) *Service registry and service broker*: Service registry and broker are smart contracts that manage IoT devices and services add process service requests and responses. During device and service registration, the service registry updates the ledger with the latest device or service information provided by the IoT device. The service registry also provides interfaces for querying IoT devices and services. When invoked, the service broker smart contract will append service requests and responses to the ledger. IoT devices and applications observe and respond to ledger updates, eventually achieving asynchronous communications between service providers and consumers.

(6) *Platform SDK*: Platform SDK provides an application programming interface (API) of the platform to IoT service and application developers. It encapsulates functions for registering devices and services, sending service requests and responses, querying platform data, etc. The goal of platform SDK is to conceal the complexity of blockchain operations from IoT devices and application developers.

(7) *Application*: An application interacts with IoT devices via the services published on the platform. For example, an application can be an industrial control system (ICS) that monitors sensor readings or a smartphone app that displays room temperature and security camera feed in a smart home environment. Additionally, applications can provide services to other IoT devices and applications on the platform.

(8) *Identity service*: Every participating actor of the proposed platform, such as an IoT device, an application, or a peer node, is recognized by its digital identity. Therefore, we need an identity service for every organization to issue, renew, and revoke those identities. The platform allows organizations of the consortium to employ their own identity services so that each organization has complete control over its assets, such as peers, IoT devices, and applications. In addition, the consortium blockchain can enforce access control to IoT services through a set of policies defined for digital identities.

(9) *Auxiliary storage*: Although IoT devices and applications mainly communicate through the blockchain, it is sometimes desirable for IoT devices to share data off-chain. The proposed platform encompasses an optional auxiliary storage system to satisfy various data storage needs. For example, the auxiliary storage can be a distributed object storage system that stores historical humidity values, a distributed message queue for sharing real-time data, such as Particulate Matter (PM) 2.5 readings, or a proxy for streaming real-time binary data, such as a camera feed. The uniform resource identifier (URI) can be passed to the data consumer via IoT services. Finally, the data may be encrypted using the data consumer’s identity to provide confidentiality.

The following sections elaborate on the necessary procedures for the IoT service platform DISP and explain how DISP takes advantage of the consortium blockchain and auxiliary storage to secure IoT systems.

### 3.1. Device and Service Registration

**Figure 2:**
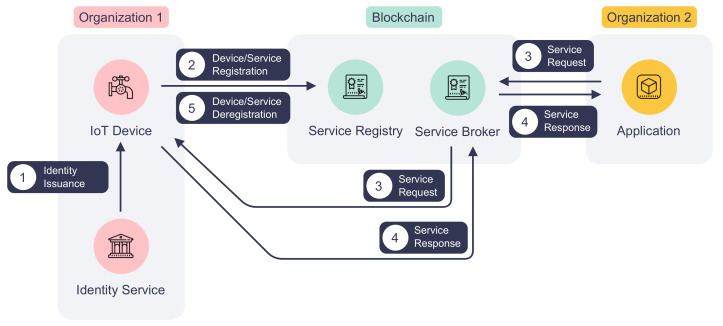
The life cycle of an IoT device and its service.

Figure 2 depicts the life cycle of an IoT device and its service on DISP. To expose functions to the network, an IoT device first must register itself and its services on the platform. Before the registration process starts, the device obtains its digital identity from the identity service of its belonging organization as marked by step ① in Figure 2. Then, it announces the device information and services by invoking the service registry smart contract, which is shown in step ② in Figure 2. If successfully validated, the device and service information will be stored on the blockchain. The registration process is required because device and service information is crucial to the authentication and access control processes, as described in Section 3.2.

(1) *Device Registration*: The first step for an IoT device to be registered DISP is receiving its digital identity from the identity service provider of its organization. Unlike many other blockchain-based IoT platforms in the literature that employ custom device identity and registration processes, our platform DISP can reuse existing digital identities issued by the organization’s PKI. Thus, organizations can follow standard procedures of signing and issuing identity certificates using an off-the-shelf certificate authority (CA). Not only can DISP benefit from the robust security of a PKI, but also participating organizations can integrate their IoT device management system easily with DISP. Consequently, IoT devices can be decommissioned by deregistering the service from the service registry and putting their identity certificates on the certificate revocation list (CRL).

Next, the IoT device can register itself by invoking the service registry with the required information. Algorithm 1 is an abstraction of the device registration process executed by the service registry smart contract. The most important fields that must be provided by an IoT device are its digital identity and the organization ID, which are used to locate a specific device on the platform. Other information, such as name, description, and last update time, are human-readable metadata for platform users. The service registry first generates the unique device ID from the digital identity. Then, it validates the caller’s identity to ensure it has sufficient permission to update the blockchain and does not impersonate other devices. Finally, the registry will serialize the provided device information and add it to the blockchain using the organization ID and device ID as the key.
**Algorithm 1** Registering a device with the service registry.**Require:** *B*:   Global state of the blockchain. *P*:   Service registry smart contract caller. $C_{dev}$:   Device’s digital identity. Must be unique within an organization. $I_{org}$: ID of the organization to which the device belongs. $N_{dev}$:  Nickname of the device. $D_{dev}$:   A short summary of the device’s function and usage. $T_{dev}$: The date and time when the last update is made to the device information.**Ensure:** Device information is saved or updated in the blockchain. 1: **if**
 WRITE∉
GetCallerPermissions
(P) **then** 2:  **return** error      ▹ Abort if caller is not allowed to update the blockchain 3: **end if** 4: $I_{dev}\leftarrow$
GenerateDeviceId
$\left(C_{dev}\right)$  ▹ Generate device ID from its digital identity 5: **if**
$\;I_{dev}\neq$
GetContractCallerId
(P) **then** 6:  **return** error           ▹ Abort if caller impersonates another device 7: **end if** 8: **if**
$\;I_{org}\neq$
GetContractCallerOrgId
(P) **then** 9: **return** error        ▹ Abort if caller impersonates another organization 10: **end if** 11:
$\;S_{dev}\leftarrow$
Serialize
$\left(I_{dev},I_{org},N_{dev},D_{dev},T_{dev}\right)$ 12:
$\;B\left[I_{org},I_{dev}\right]\leftarrow S_{dev}$ 13: **return**
$\;S_{dev}$


(2) *Service Registration*: Registered IoT devices publish their services on the network via the service registry smart contract. Such a service can measure a room’s humidity or setting a refrigerator’s temperature. An IoT device may declare multiple services on the blockchain by repeating the registration process. Similar to device registration, an IoT device is required to provide service information to the service registry for each service. The service registration process is outlined in Algorithm 2. The service name, device ID, and organization ID identify a unique service in the network. Therefore, each service has a unique service name on a device. The service version number and last update time are useful fields for application developers and system auditors to keep track of service updates. The description field offers a summary of the service and its usage. Besides identity and permission validation, the service registry will also check if the provided device is already registered on the platform. If there is no issue, it will write the serialized service information to the blockchain with the organization ID, device ID, and service name as the key. In accordance with the device decommission process, the service registry also allows IoT devices to deregister their services by calling the corresponding function in the smart contract, as shown in step ⑤ in Figure 2.
**Algorithm 2** Registering a service with the service registry.**Require:** *B*:   Global state of the blockchain. *P*:    Service registry smart contract caller. $N_{srv}$:   Name of the service. Must be unique among all services of the same device. $I_{dev}$: ID of the device that provides the service. $I_{org}$:    ID of the organization to which the device belongs. $V_{srv}$:    Version number of the service. $D_{srv}$:   A short summary of the service’s function and usage. $T_{srv}$: The date and time when the last update is made to the service information.**Ensure:** Service information is saved or updated in the blockchain. 1: **if**
 WRITE∉
GetCallerPermissions
(P) **then** 2:  **return** error      ▹ Abort if caller is not allowed to update the blockchain 3: **end if** 4: **if**
$\;I_{dev}\neq$
GetContractCallerId
(P) **then** 5:  **return** error          ▹ Abort if caller impersonates another device 6: **end if** 7: **if**
$\;I_{org}\neq$
GetContractCallerOrgId
(P) **then** 8:  **return** error         ▹ Abort if caller impersonates another organization 9: **end if** 10: **if**
GetDevice
$(I_{org},I_{dev})=\emptyset$ **then** 11:  **return** error               ▹ Abort if device is not registered 12: **end if** 13:
$\;S_{srv}\leftarrow$
Serialize
$\left(N_{srv},I_{dev},I_{org},V_{srv},D_{srv},T_{srv}\right)$ 14:
$\;B\left[I_{org},I_{dev},N_{srv}\right]\leftarrow S_{srv}$ 15: **return**
$S_{srv}$


### 3.2. Authentication and Access Control

**Figure 3:**
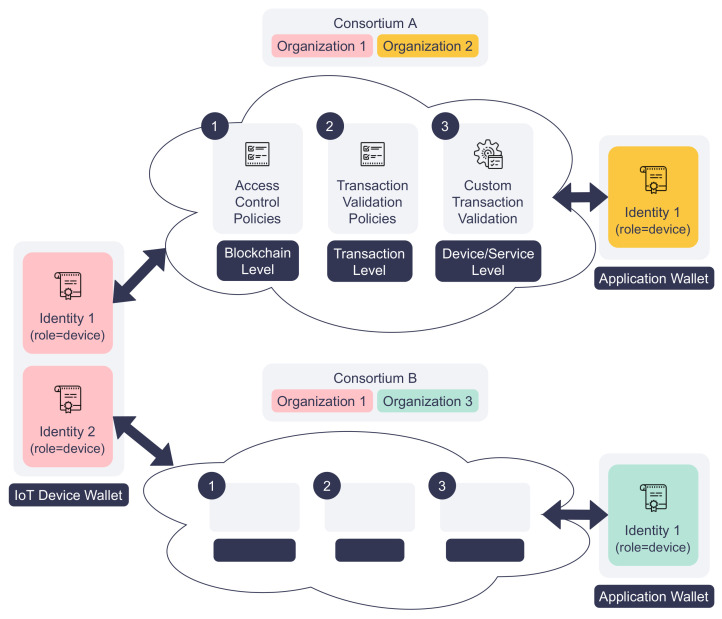
The proposed multi-layered access control model.

DISP enforces authentication and access control to prevent unauthorized access. As stated in Section 3.1, the platform’s authentication and access control rely on digital identities issued by the identity service. All the platform actors, such as IoT devices and applications, must have at least one valid digital identity to interact with the blockchain. The smart contracts will verify the identity of calling actors to see if they have the necessary permissions to read or write the distributed ledger. To enable cross-organization operations, the actors can possess multiple digital identities issued by different identity services, as depicted in Figure 3. Each identity, including its certificate cert and private key prikey, is stored in a blockchain wallet *W*. Therefore, the platform allows an IoT device to register its service in multiple organizations using different identities stored in the same wallet, i.e., $W=\{\left(cert_{i},prikey_{i}\right)|i=1,2,\ldots ,N\}$.

DISP enforces more granular access control via a multi-layer RBAC model, as shown in Figure 3. In this model, the digital identity of each actor is assigned a role when it is created. The first layer, the blockchain-level access control policies, define which organizations and roles can query or update the ledger using smart contracts. By default, actors of a participating organization can see all registered IoT devices and services, but only the actors with the “writer” roles have the right to register or update them. At the next layer, the transaction level, service registry, and service broker smart contracts can be attached with transaction validation policies. These policies tell which organizations and actors must sign the service request transaction for it to be valid. Finally, the smart contracts enforce the last layer of access control for each IoT device, service, service request, and service response. The smart contracts check the caller’s identity to ensure that only the service-owning IoT device can update the device and service information and respond to service requests.

### 3.3. Service Request and Response

**Figure 4:**
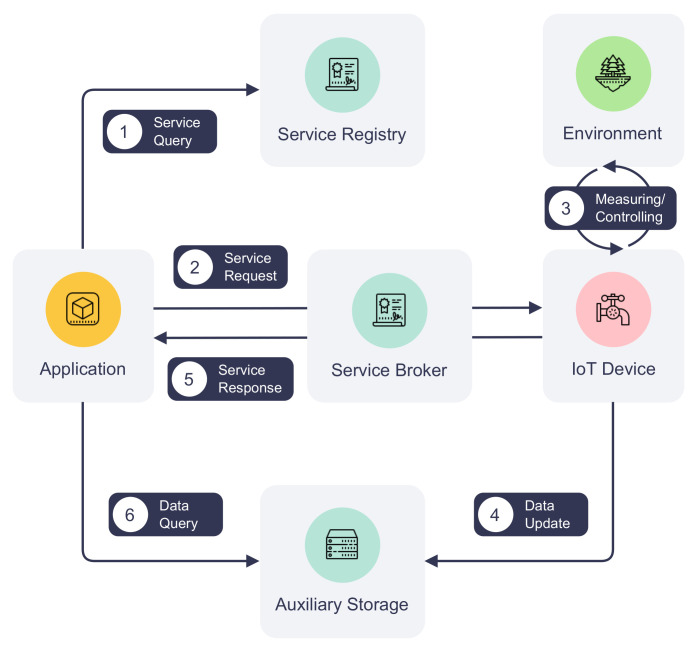
Process of communication between an IoT device and application.

The consortium blockchain provides opportunities for IoT networks to become decentralized and distributed. It also enables sharing of IoT infrastructure among organizations within a consortium in a scalable and reliable manner. Leveraging the advantages of consortium blockchain, we propose an IoT communication process that utilizes the blockchain network as the communication channel. In this process, data exchange between parties is achieved in service requests and responses facilitated by the service broker smart contract. Figure 4 provides a more detailed depiction of steps ③ and ④ of Figure 2. It illustrates the communication process between an IoT device and an application, including querying available services, requesting services, responding to service requests, and retrieving data.

(1) *Service querying*: To find out a service’s availability, an application can query the service registry before initiating a service request, as illustrated by step ① in Figure 4. Given the service name, device ID, and organization, the service registry returns information about the service to the application, such as the service version and last update time. Although this step is not mandatory, an application is recommended to refresh service information periodically to stay updated on the service’s information and look for alternative services when the service is unavailable. Additionally, the last update time of service helps decide the best time to refresh such information.

(2) *Service request and processing*: To communicate with an IoT device on DISP, an application creates a service request first. Every service request is identified by a universally unique identifier (UUID) generated by the application. The request ID must be unique among all requests on the platform. Information about the requested service, such as the service name, is required so that the service-providing IoT device can retrieve the request from the blockchain. The request also contains the request creation time used for request deduplication and auditing purposes. The request body is represented by the method and arguments fields where the action and its optional arguments are defined.

Next, the application submits the service request to the service broker, which will validate the request and create a blockchain transaction. Algorithm 3 illustrates the service request processing at a high level. The service broker smart contract first verifies the contract caller’s permissions to ensure it can update the blockchain. Additionally, it validates the requested service and skips duplicated requests. The request is then serialized and saved in the blockchain through a blockchain transaction. Once the transaction is validated and endorsed by the network and written to the ledger, the service-providing IoT device will be notified by the network that its service has been requested. An IoT device can then fetch request information from the blockchain via the service broker and interact with its environment as instructed by the request. These processes are depicted in steps ② and ③ in Figure 4.
**Algorithm 3** Creating a service request using the service broker.**Require:** *B*:   Global state of the blockchain. *P*:   Service broker smart contract caller. $I_{req}$:    UUID of the request. $T_{req}$: Date and time when the request is created. $N_{srv}$:   Name of the requested service. $I_{dev}$:    ID of the device that provides the service. $I_{org}$: ID of the organization to which the device belongs. $M_{req}$:  Request method. $A_{req}$:  Optional arguments of the request.**Ensure:** Service request information is saved in the blockchain. 1: **if**
 WRITE∉
GetCallerPermissions
(P) **then** 2:  **return** error      ▹ Abort if caller is not allowed to update the blockchain 3: **end if**4: **if**
GetService
$(I_{org},I_{dev},N_{srv})=\emptyset$ **then** 5:   **return** error          ▹ Abort if requested service is not registered 6: **end if** 7: **if**
GetServiceRequest
$(I_{req})\neq \emptyset$ **then** 8:     **return** error              ▹ Abort if service request already exists 9: **end if** 10:
$S_{req}\leftarrow$
Serialize
$\left(I_{req},T_{req},N_{srv},I_{dev},I_{org},M_{req},A_{req}\right)$ 11:
$B\left[I_{req}\right]\leftarrow S_{req}$ 12: NotifyEvent
$\left(S_{req}\right)$ 13: **return**
$S_{req}$


(3) *Service response and data retrieval*: IoT devices can respond to service requests asynchronously whenever they finish processing them. The process of replying to a service request is shown in steps ④ to ⑥ in Figure 4. An IoT device starts by creating the service response. Besides the UUID of the corresponding request, the response also contains fields about response creation time, a custom status code indicating the status of processing, and an optional value to be returned to the requester. The device may also store operational data in the auxiliary storage and leave a pointer to the data in the return value under situations where the volume or time sensitivity of the data cannot be met by the blockchain. Details of the auxiliary storage are further discussed in Section 3.4.

Following its creation, the service response is sent by the IoT device to the service broker, which creates another blockchain transaction. Algorithm 4 depicts the service response creation process in the service broker. Similar to the service request creation, the broker validates the caller’s identity and the existence of the request. It also prevents service impersonation by matching the caller’s identity against the identity of the requested service. The service response update transaction will undergo the same process of validation and endorsement as the service request transaction and eventually be appended to the distributed ledger. By listening to transaction events on the blockchain, an application can act upon the completion of its request, e.g., retrieving the response from the blockchain. Furthermore, if the return value contains a pointer to data in the auxiliary storage, the application can perform additional storage operations to retrieve the data.
**Algorithm 4** Responding to a service request using the service broker.**Require:** *B*:   Global state of the blockchain. *P*:   Service broker smart contract caller. $I_{req}$:    UUID of the request to which the response answers. $T_{res}$: Date and time when the response is created. $U_{res}$:   The request processing status. $R_{res}$:   Optional value to be returned to the service request sender.**Ensure:** Service request information is saved in the blockchain. 1: **if**
WRITE∉
GetCallerPermissions
(P) **then** 2:  **return** error      ▹ Abort if caller is not allowed to update the blockchain 3: **end if** 4: **if**
GetService
$(I_{org},I_{dev},N_{srv})=\emptyset$ **then** 5:  **return** error             ▹ Abort if requested service is not registered 6: **end if** 7: $S_{req}=$
GetServiceRequest
$\left(I_{req}\right)$ 8: **if**
$S_{req}=\emptyset$ **then** 9:  **return** error               ▹ Abort if service request does not exist 10: **end if** 11: **if**
GetDeviceId
$\left(S_{req}\right)\neq$
GetContractCallerId
(P) **then** 12:  **return** error            ▹ Abort if caller impersonates another device 13: **end if** 14: **if**
GetOrganizationId
$\left(S_{req}\right)\neq$
GetContractCallerOrgId
(P)
**then** 15:  **return** error      ▹ Abort if caller impersonates another organization 16: **end if** 17:
$S_{res}\leftarrow$
Serialize
$\left(I_{req},T_{res},U_{res},R_{res}\right)$ 18:
$B\left[I_{req}\right]\leftarrow \left\{S_{req},S_{res}\right\}$ 19: NotifyEvent
$\left(S_{res}\right)$ 20: **return**
$S_{res}$


### 3.4. Data Storage

**Figure 5:**
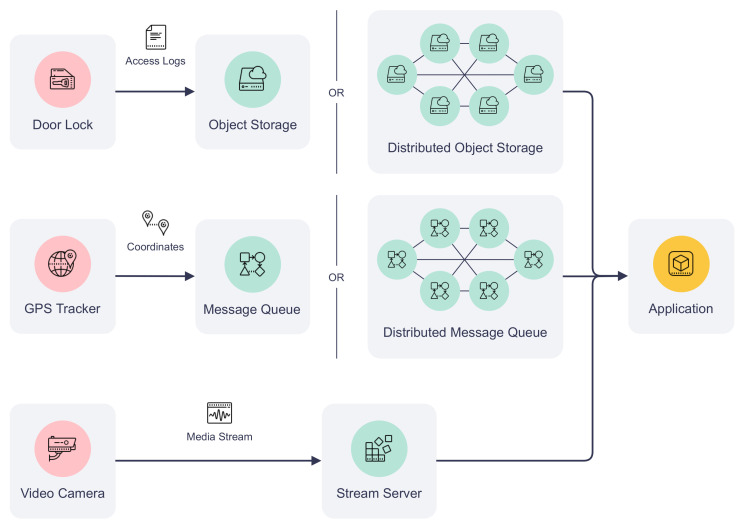
Auxiliary storage types and applications.

Due to blockchain’s transaction size, throughput, and latency limitations, it is often preferable to store IoT-generated data off-chain. To meet the storage requirements of different IoT applications, auxiliary storage can be included to complement the data exchange on the platform. Figure 5 demonstrates how auxiliary storage facilitates IoT devices to pass the data to an application. The storage system can be composed of different types of data storage depending on the communication requirements. For example, non-real-time structured data can be stored in a cloud, such as AWS S3, or a distributed object storage, such as the InterPlanetary File System (IPFS). IoT device logs and historical sensor readings fall into this category. Meanwhile, real-time sensor data (e.g., instantaneous geolocation coordinates) can be published to message queues such as Apache Kafka for efficient data delivery. Finally, media streaming servers can be included in the auxiliary storage to transcode, cache, and stream multimedia captured by IoT devices to their users. Examples of these data types are surveillance camera feed and audio data collected by an acoustic gunshot detection system. Each type of storage can be offered by a single organization of the consortium or hosted by multiple organizations collaboratively. The design of data storage is out of the scope of this paper.

## 4. Implementation and Case Studies

This section dives into the implementation details of the proposed platform. We further showcase the capabilities and usefulness of the platform with two real-world IoT applications. For the sake of research reproducibility, the source code of our implementation, the exemplary case study applications, and all the scripts for testbed setup and benchmarking are made available online (Please refer to the Data Availability Statement).

### 4.1. Platform Implementation

Considering the feature richness and development support, we selected Hyperledger Fabric [22] as the consortium blockchain platform for our IoT service platform implementation. Thanks to its modular architecture and plug-and-play nature, Hyperledger Fabric has been widely used in industrial environments. Our implementation takes advantage of Hyperledger Fabric’s components to realize the core functionality of DISP in the following aspects:

(1) We incorporated device and user identities into the blockchain using Hyperledger Fabric’s membership service provider (MSP). Each organization on the platform has its dedicated MSP, which translates the identities into roles and privileges of the blockchain. Thus, the platform can authenticate invocations to smart contracts using registered identities.

(2) Hyperledger Fabric defines various policies agreed upon by the consortium members, or channel members in Hyperledger Fabric’s terminology, for infrastructure management. In our implementation, we limit IoT devices and users to only submitting transactions or querying the ledger using ACL. We also restrict which organizations must approve or endorse the transactions with smart contract endorsement policies. In addition, smart contracts also limit access to write operations, such as responding to service requests to the device and service owners by checking the caller’s identity.

(3) The smart contracts of the proposed platform were implemented using Fabric contract API in the Go programming language. All smart contracts are packaged in one chaincode, a container for smart contracts, so that they share the same world state. In addition, we created SDK for Go, Java, JavaScript, and TypeScript programming languages to simplify application development for DISP.

(4) Client communications with the blockchain are simplified using the Hyperledger Fabric gateway. Instead of directly interacting with the blockchain network and managing the complexity of transaction proposal, endorsement, and commission, IoT devices and users delegate most of the heavy-lifting operations to a gateway component running on peer nodes. This improvement is essential for devices that are energy-constrained or low on computing power.

### 4.2. Case Study: Parrot

**Figure 6:**
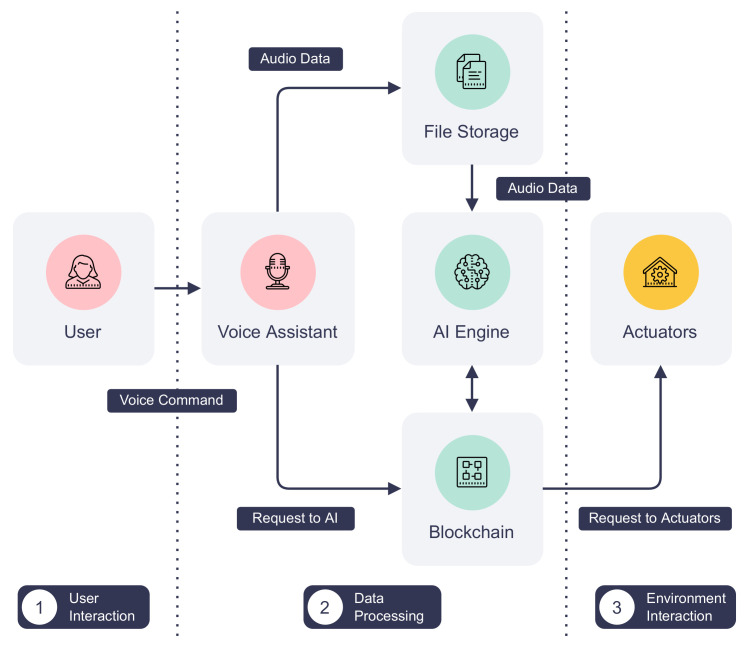
The architecture and data flow of Parrot.

Parrot is a voice assistant for the smart home lighting system implemented using the proposed IoT service blockchain. It enables touchless control of home lights using voice commands, such as “Parrot, turn on the kitchen light”. The architecture of Parrot is shown in Figure 6. The workflow starts with a user issuing a voice command in a given format to a smart speaker. A microphone on the smart speaker continuously listens in the background but only begins recording voice commands on wake words, “Parrot” in this case. It also determines the duration of the recording and stops when the command ends. This is done using an onboard wake word detection engine, Porcupine [42], and the voice activity detector provided by WebRTC [43]. Then, the smart speaker adds the recording to a private decentralized file storage network, implemented by IPFS, and calls the service exposed by a remote voice AI engine to the IoT service platform. Next, the AI engine is notified by the blockchain and retrieves the user’s audio recording from IPFS. The data are then fed into Rhino [44], a speech-to-intent engine that decodes voice commands and extracts the location of the light and actions to perform. Finally, the AI engine sends requests for turning on or off to the corresponding actuator, i.e., the smart light, via the blockchain.

The main advantage of this model is that all communications are securely backed by the proposed platform DISP. Compared to traditional IoT systems, actors of this system publish their services only to the blockchain instead of exposing them using other insecure communication protocols. Moreover, all processes involved are transparent to users since service activities are recorded on the immutable blockchain. Blockchain transactions leave an audit trail that is invaluable to incident response and forensic investigation when a problem arises. Regarding user privacy, the network operator can isolate different user groups using separate Hyperledger Fabric channels. Audio recordings of the user can also be set to expire automatically by unpinning and garbage collecting them from the storage network.

### 4.3. Case Study: Crystal Ball

Surveillance cameras are widely deployed nowadays. However, they are often vulnerable to hackers or malware due to poor security design and improper configuration. An unprotected surveillance camera can seriously threaten user privacy as the video footage may be leaked to unauthorized parties. For example, Insecam (http://www.insecam.org (accessed on 22 September 2022)) is a live camera directory that allows visitors to view live streams from thousands of unprotected public cameras as of May 2022, and the number of exposed cameras is still growing. Hackers can also use a compromised camera in other cyber crimes, e.g., to form a botnet and initiate distributed denial-of-service (DDoS) attacks [45].

**Figure 7:**
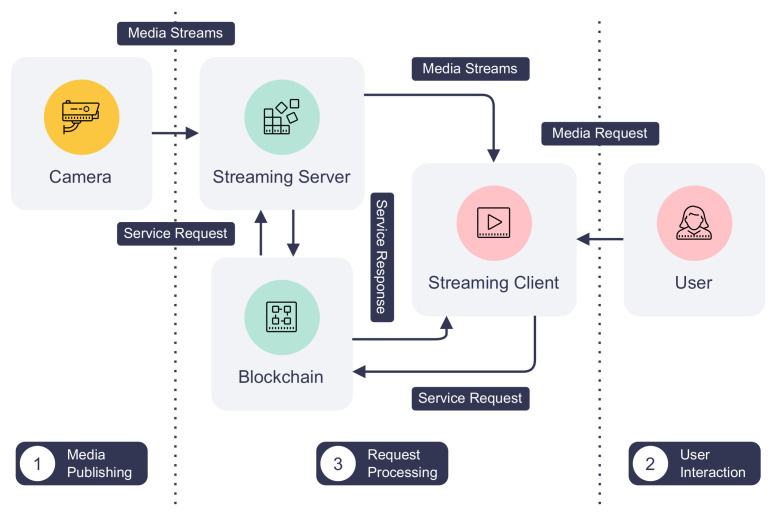
The architecture and data flow of Crystal Ball.

We have built a blockchain-based secure surveillance streaming system called Crystal Ball using DISP. Figure 7 depicts the architecture and workflow of Crystal Ball. A camera in the Crystal Ball system does not serve its video and audio feeds on an open network port. Instead, it publishes them to a secure streaming server that streams camera feeds only to users with correct access tokens. Meanwhile, the streaming server creates an IoT service that generates and distributes one-time session access tokens to authorized users on the blockchain. Finally, users can request access tokens and watch live streams from an Android streaming client.

Crystal Ball eliminates the need for exposing camera feeds from the capturing device. Unnecessary ports can now be closed to reduce the attack surface of the camera. Furthermore, Crystal Ball protects camera feeds from unauthorized access using device identities and access tokens. As access tokens are one-time only and tied to each session, a malicious user cannot reuse previous tokens even if they are leaked. Finally, an administrator can easily log and analyze camera accesses using transaction history and decommission cameras that are compromised by invalidating their device identities.

## 5. Evaluation and Discussion

In this section, we first present the experiment settings under which we evaluated the proposed IoT service platform DISP. Our evaluation focuses on two core configuration parameters of the blockchain, transaction batch size, and batch timeout, to discover how blockchain configuration affects the performance of DISP. We then examine the transaction performance overhead introduced by additional IoT device connections. Transaction throughput, transaction latency, and system resource utilization are measured in each test as the performance indicators of the platform. Finally, we discuss DISP’s security and privacy and its impact on IoT systems.

### 5.1. Experimental Setup and Methodology

**Figure 8:**
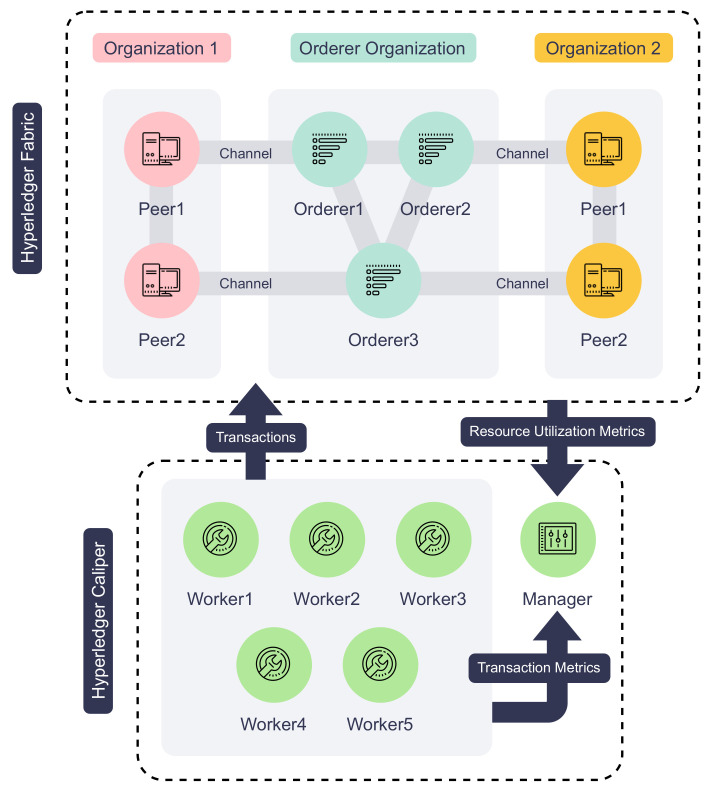
IoT service platform testbed architecture.

Figure 8 illustrates the architecture of our testbed and Table 2 details the hardware and software configuration of each machine used in our experiments. The proposed IoT service platform ran on a multi-node Hyperledger Fabric blockchain comprising two peer organizations and one orderer organization. Each peer organization contains two peer nodes. The orderer organization contains three Raft orderer nodes, and all run Hyperledger Fabric version v2.4.3. All the organizations form a single consortium, and transactions are executed and ordered in a single channel. The channel enforces the “MAJORITY” endorsement policy, which means the two peer organizations must both endorse the transaction for it to be committed to the blockchain. Then, the IoT service blockchain chaincode is installed on all the peer nodes. To resemble a multi-organizational environment, we deployed the testing blockchain on bare-metal servers across two Chameleon Cloud [46] data centers. Compared to deploying all nodes to the same data center, our setup introduces a network latency of around 30 ms between consortium parties to reflect a network environment of a real-world blockchain network.

Apart from the Hyperledger Fabric blockchain nodes, six virtual machines were employed to establish the Hyperledger Caliper [47] benchmarking environment. Hyperledger Caliper is a blockchain benchmarking tool that generates synthetic transaction workloads and measures the performance of the system under test (SUT). Our benchmarking environment is composed of a Caliper manager and five Caliper workers. The manager distributes workload parameters to the workers, synchronizes workers during each test, collects evaluation results, and generates human-readable reports. Each of the five workers starts two client connections to the blockchain, which simulates ten IoT devices that provide or request services on the proposed platform. These workers execute the workload scripts that call the smart contracts and generate transactions guided by the fixed-load rate control strategy. This strategy guides the workers to send the transactions or queries at a dynamic rate such that the number of incomplete transactions or queries in the SUT always stays under a given value. In our experiments, the workers collectively send 2000 transactions or queries under a fixed load of 100 transactions/queries. Finally, updating the same device, service, service request, or response in a single test is avoided in order to work around mvcc_read_conflict errors in Hyperledger Fabric. Compared to the blockchains such as Ethereum that have a more serialized approach to transaction processing, Hyperledger Fabric employs lock-free optimistic concurrency. To prevent the double-spending problem, Hyperledger Fabric will only process one of all transactions that modify the same data and reject the rest in the same block [48].

The core metrics inspected in each test include peak read/transaction throughput and average read/transaction latency. According to the Hyperledger Blockchain Performance Metrics white paper [49], a read operation does not change the ledger state, while a transaction operation involves ledger updates. The latency $L_{read}$ of a read operation measured in seconds is defined as:

(1) $$L_{read}=t_{response}-t_{submit},$$

where $t_{submit}$ is the read request submission time and $t_{response}$ is the time at which the reply is received. The throughput $W_{read}$ of read operations measured in RPS is defined as:

(2) $$W_{read}=\frac{N_{read}}{T_{read}},$$

where $N_{read}$ is the total number of read operations completed in time $T_{read}$.

Transaction throughput and latency are measured differently from the read throughput and latency since the confirmation time of blocks must be considered in transaction operations. The transaction latency $L_{tx}$ in seconds is defined as:

(3) $$L_{tx}=t_{confirm}-t_{submit},$$

where $t_{confirm}$ is the time at which the transaction is confirmed by the network given a network threshold (e.g., 90% of the network), and $t_{submit}$ is the time when the transaction is submitted by the client. The transaction throughput $W_{tx}$ measured in TPS is defined as:

(4) $$W_{tx}=\frac{N_{tx}}{T_{tx}},$$

where $N_{tx}$ is the total number of committed transactions at all nodes of the network in time $T_{tx}$.

System resource utilization metrics are also measured during each test. They include average CPU and memory usage, total data sent to or received from the network, and total data read from or written to the disk. These metrics are polled and aggregated by Prometheus [50] periodically from each Hyperledger Fabric node and reported to the Caliper manager. The read/transaction throughput and latency of the blockchain and the above resource utilization metrics collectively indicate how well our proposed platform performs under different blockchain configurations. They also provide valuable information on optimizing the blockchain for various use cases.

### 5.2. Performance vs. Batch Size

We first examine the impact of transaction batch size on the performance of DISP. Three parameters constrain the batch size in the Hyperledger Fabric blockchain: maximum message count $N_{max}$, preferred maximum bytes of messages $S_{soft}$, and absolute maximum bytes of messages $S_{hard}$. Therefore, the batch size configuration directly controls the number of messages $N_{msg}$ that can be batched into a block. When the size of each message, or transaction data, is relatively small, $N_{msg}$ is primarily limited by $N_{max}$. Otherwise, $N_{msg}$ will mostly be limited by $S_{soft}$ unless there are many huge messages whose sizes exceed $S_{hard}$.

In our experiments, we intend to control the batch size solely with $S_{soft}$. So we assign large constant values to $N_{max}$ and $S_{hard}$ to ensure blocks always reach $S_{soft}$ first. Then, we set $S_{soft}$ to 128 KB, 256 KB, 512 KB, 1 MB, 2 MB, 4 MB, and 8 MB, respectively, and evaluate the smart contract operations of DISP. Device registration and deregistration transaction throughput and latency are omitted from the results since the workers only start ten blockchain clients for the tests. As each IoT device binds to one client identity, the workers are limited to registering or deregistering ten devices in total. Therefore, insufficient transactions can be evaluated for the above two operations to report their accurate throughput and latency results, and we exclude these operations from the result analysis. However, we will revisit these operations and their performance in later experiments.

**Figure 9:**
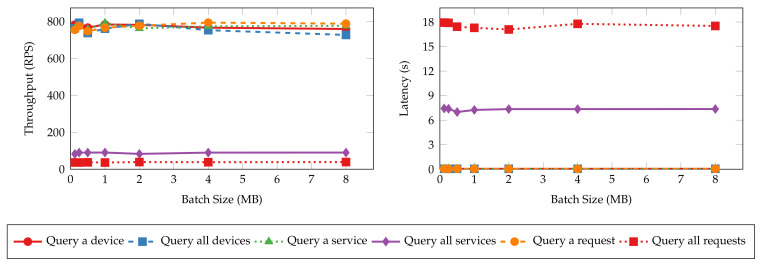
Throughput and latency of read operations with varying batch sizes.

**Figure 10:**
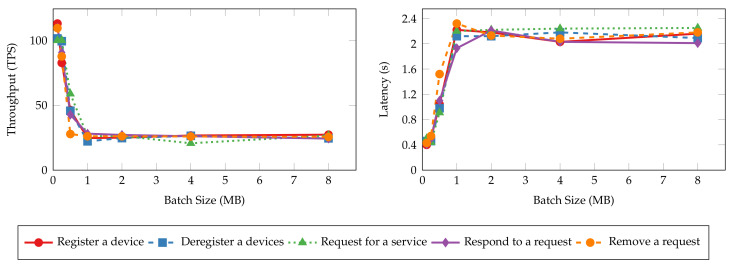
Throughput and latency of transaction operations with varying batch sizes.

Figure 9 and Figure 10 present the throughput and latency of read and transaction operations under various batch sizes. The throughput and latency of all read operations stay nearly unchanged as batch size increases. This is because read operations in Hyperledger Fabric are not sent to the ordering service for validation and committing to the ledger [51]. Thus, batch size configuration has no impact on the performance of read operations. On the other hand, the size of messages has more impact on the performance metrics. The “query all requests” and “query all services” operations have lower throughput and higher latency values as the messages returned to the clients are significantly larger than the singular queries (e.g., “query a device”) and device queries. It can take more time and bandwidth for the peers to process and transmit such messages, resulting in lower performance. In contrast, the performance metrics of transaction operations are tied more closely to batch sizes. The throughput and latency degrade quickly from 100 TPS and 0.4 s to 25 TPS and 2.4 s as the batch size increases until it reaches 1 MB since larger blocks need more time to generate and dispatch. Further increasing the batch size will not worsen the performance.

**Figure 11:**
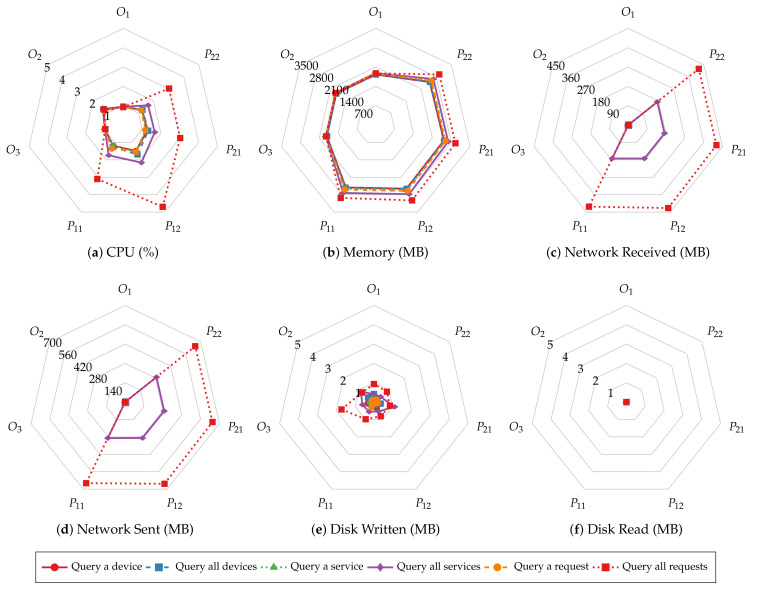
The average CPU usage (**a**), average memory usage (**b**), total data received from the network (**c**) and sent to the network (**d**), total data written to disk (**e**) and read from disk (**f**) of orderer1 (*O*_1_), orderer2 (*O*_2_), oderer3 (*O*_3_), org1 peer1 (*P*_11_), org1 peer2 (*P*_12_), org2 peer1 (*P*_21_), and org2 peer2 (*P*_22_) during read operations when $S_{soft}$ = 2 MB.

**Figure 12:**
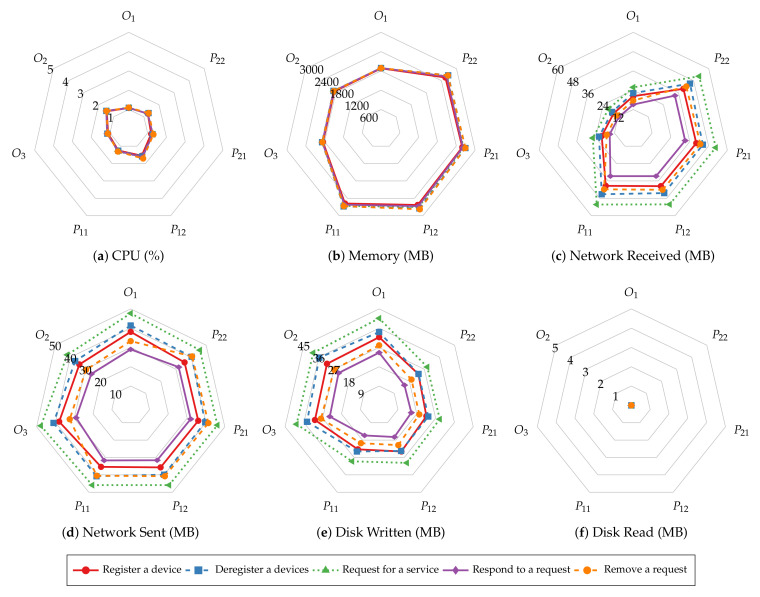
The average CPU usage (**a**), average memory usage (**b**), total data received from the network (**c**) and sent to the network (**d**), total data written to disk (**e**) and read from disk (**f**) of orderer1 (*O*_1_), orderer2 (*O*_2_), oderer3 (*O*_3_), org1 peer1 (*P*_11_), org1 peer2 (*P*_12_), org2 peer1 (*P*_21_), and org2 peer2 (*P*_22_) during read operations when $S_{soft}$ = 2 MB.

Before investigating batch size’s impact on system resource utilization of smart contract operations, we first explore the similarities in resource utilization patterns of different operations. Figure 11 and Figure 12 present the average CPU, average memory, total network, and total disk usage of every host of the test Hyperledger Fabric network with 2 MB batches. The results show that the peer nodes are more utilized than the orderer nodes under most workloads. Moreover, the utilization levels are even on the same type of nodes. Regardless of the specific operation performed, all the read operations show similar resource usage patterns, and so are the transaction operations. Meanwhile, the transaction operations involve more incoming network traffic and disk writes than the read operations, and the operations that consume and produce larger messages require more CPU and memory. Finally, disks are rarely read in all the tests due to ledger data being loaded to memory before the measurements begin.

**Figure 13:**
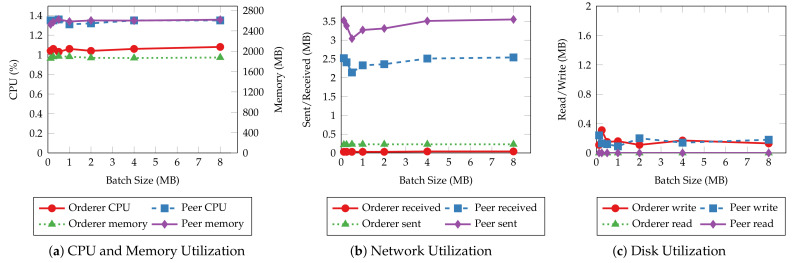
The average CPU and memory usage (**a**), total data received from and sent to the network (**b**), total data written to and read from disk (**c**) of orderer and peer nodes for handling “querying a service request“ operations with varying batch sizes.

**Figure 14:**
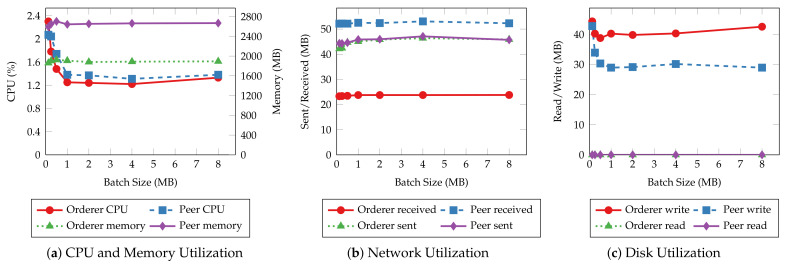
The average CPU and memory usage (**a**), total data received from and sent to the network (**b**), total data written to and read from disk (**c**) of orderer and peer nodes for handling “requesting for service“ operations with varying batch sizes.

The same resource utilization patterns are also observed in other tests with varying batch sizes. Therefore, we only present the results of the “request for a service” and “query a request” operations to demonstrate the relationship between resource utilization and batch size. Figure 13 and Figure 14 depict the CPU, memory, network, and disk usage of each test given each batch size. For read operations such as querying a single service request, there is slight fluctuation in system resource usage as the batch size increases, similar to their throughput and latency. This result aligns with the fact that those operations do not go through the ordering process. On the other hand, transaction operations incur a higher CPU utilization and more disk writes when the batch size is small. It is clear that with smaller batch sizes, the network needs to create more blocks to commit the same number of transactions, hence, more overhead in the data to be written to disk and more computing power required to complete that. The network usage does not change significantly with batch size because the sizes of incoming and outgoing messages remain the same regardless of batch size.

### 5.3. Performance vs. Batch Timeout

**Figure 15:**
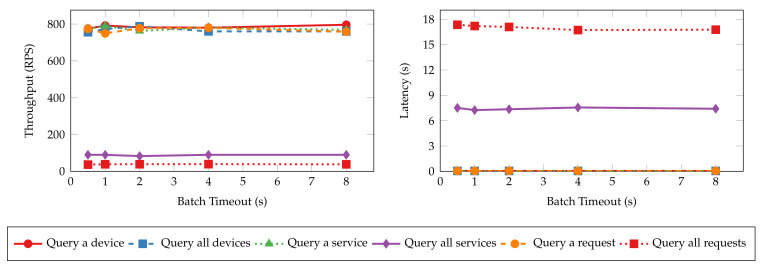
Throughput and latency of read operations for varying batch timeouts.

**Figure 16:**
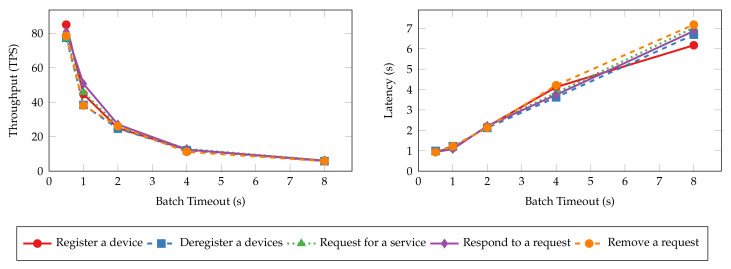
Throughput and latency of transaction operations for varying batch timeouts.

Batch timeout ($T_{timeout}$) is another essential configuration that controls the generation of blocks. It is the maximum time to wait before creating a new block after the first transaction arrives at the ordering service. To estimate the impact of batch timeout on the performance of the proposed system, we perform the same experiments as the ones in the previous section, with batch timeout set to 500 ms, 1 s, 2 s, 4 s, and 8 s, respectively. The throughput and latency of the read and transaction operations are shown in Figure 15 and Figure 16, respectively. Similar to the results of batch size tests, the throughput and latency of read operations are unaffected by the change of batch timeout because the ordering service does not process such operations. Transaction operations show a logarithmic decrease in throughput and a linear increase in latency regarding batch timeout. Blocks wait longer to be created when timeout is large, and the commit of transactions is delayed as a result. However, setting a large timeout also allows more transactions to be batched into the same block, mitigating the decrease in throughput.

**Figure 17:**
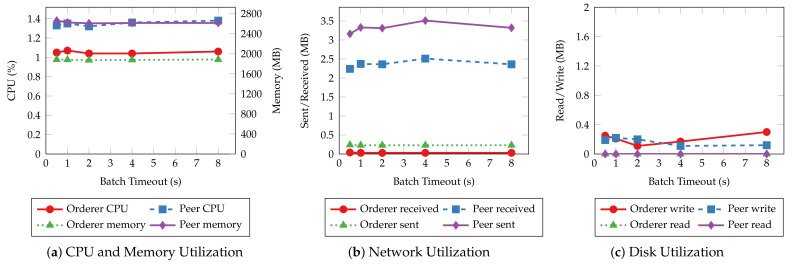
The average CPU and memory usage (**a**), total data received from and sent to the network (**b**), total data written to and read from disk (**c**) of orderer and peer nodes for handling “querying a service request” operations with varying batch timeouts.

**Figure 18:**
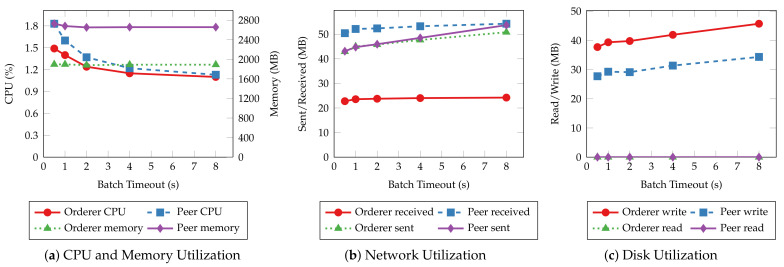
The average CPU and memory usage (**a**), total data received from and sent to the network (**b**), total data written to and read from disk (**c**) of orderer and peer nodes for handling “requesting for service” operations with varying batch timeouts.

Figure 17 and Figure 18 depict the average system resource utilization of orderer and peer nodes for the tested batch timeouts. Similar to the previous experiments, read operations exhibit consistent resource usage given different batch timeouts. Transaction operations, on the other hand, consume more orderer and peer CPU for small batch timeouts. The CPU usage decreases in the same fashion as the throughput when the timeout increases. A steady climb in the outgoing network traffic and disk write can also be observed as the timeout increases. Other resources, such as memory and incoming network traffic, are much less affected by the batch timeout.

### 5.4. Performance vs. Connection Size

A real-world IoT network can be composed of hundreds of devices that actively provide services simultaneously. The increase in device connections inevitably affects the responsiveness of our proposed platform. Therefore, our final evaluation focuses on the impact of client connection size on DISP’s performance and system resource utilization. We created 2000 client connections to the platform using Hyperledger Caliper in order to simulate the situation where a massive number of IoT devices communicate through the platform concurrently. Then, we rerun the tests with the batch size configured to 2 MB and batch time to 2 s. The results are compared with the previous results where ten client connections were employed to discover the performance and resource utilization overhead of DISP with a massive number of connections.

**Figure 19:**
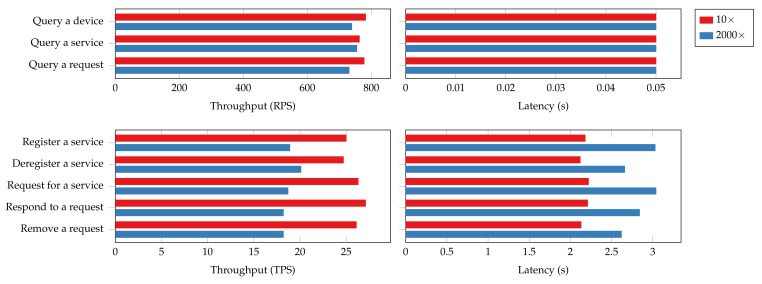
Throughput and latency of read and transaction operations for 10 client connections (10×) and 2000 client connections (2000×).

Figure 19 presents the throughput and latency of certain operations under two connection size settings. Batch queries such as “query all services” are excluded from the evaluation. This is because the numbers of devices or services they query are different under the two testing scenarios, which makes the results incomparable. For the read operations, the throughput experiences a 5% to 6% decrease in the 2000 connections scenario while the latency remains the same. For the transaction operations, the throughput suffers a 30% degradation, and the latency increases by 25% to 38%. These notable overheads result from massive clients collecting and submitting endorsements from peers. The more connection established to the peers and orderers, the more congestion there will be hindering transaction processing. Therefore, a recommendation for performance tuning would be to limit the number of client connections to each peer.

**Figure 20:**
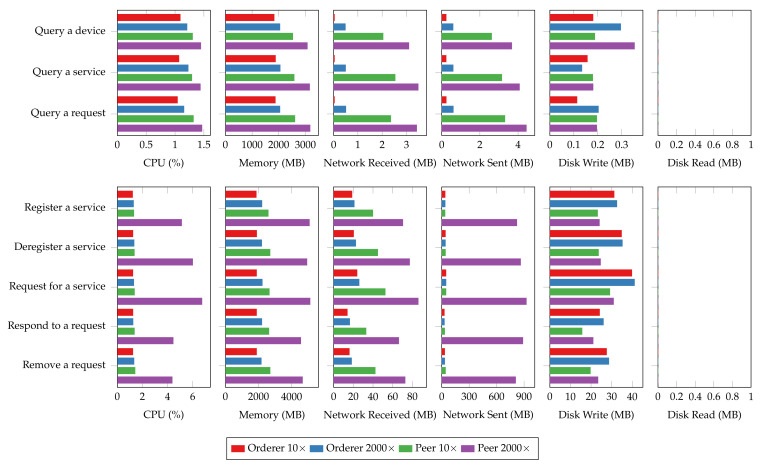
Average system resource utilization of orderer and peer nodes for 10 client connections (10×) and 2000 client connections (2000×).

The system resource utilization also rises in the 2000-connection scenario, as shown in Figure 20. The most significant increases in relative resource utilization happen in networking, where a hundred-time growth in outgoing network traffic can be observed. A drastic usage increase in CPU and memory can also be observed for both read and transaction operations. Additionally, transaction operations are more fickle to changes in connection size compared to read operations, and so do peer nodes than orderer nodes. The disk usage, however, does not vary too much with the connection size. The overhead observed in system resource utilization could be attributed to the connection overhead as the nodes have to maintain the connections from each client, verify client identities, and secure mutual communications using encryption. The results also imply that the memory and network can quickly become the bottleneck of the blockchain platform for large IoT networks.

### 5.5. Performance Comparison with Related Work

We compare the evaluation setup and performance of our proposed platform with those in other related studies in the literature. The comparison results are shown in Table 3. Unfortunately, many related studies neither detail the settings for evaluation nor explain their measurements. Therefore, we mark the missing data as “Not available”. For the studies that provide performance evaluation results, we capture the average throughputs and latencies for read and transaction operations. The performance results of some platforms were measured under different evaluation setups and obtained with different methods (e.g., round-trip time (RTT) instead of latency). Nevertheless, these results are valuable to evaluate related blockchain-based IoT platforms at a high level. Finally, we include our solution’s average read and transaction throughputs and latencies, excluding the results of batch queries when the batch size is set to 2 MB and timeout set to 2 s.

As it is shown in Table 3, our proposed platform DISP achieves much better throughput and latency for read operations than the other platforms while providing comparable throughput and latency for transaction operations. Our evaluation setup is also closer to a real-world distributed system setup than the others, as a geographically distributed consortium with multiple organizations was simulated. Moreover, the scalability of DISP was evaluated using different client or connection sizes, while most of the other studies do not provide such information. In summary, the extensive evaluation demonstrates a favorable performance result of our solution compared to the other solutions in the literature.

### 5.6. Security and Privacy

It is essential that our proposed platform DISP provides a secure environment for IoT devices and applications by leveraging blockchain technologies and modern cryptography. It is also important that we preserve user and data privacy wherever needed. In this section, we examine the security and privacy of DISP from the perspectives of blockchain, auxiliary storage, and IoT devices.

(1) *Blockchain security and privacy.* Ferrag et al. [52] presented a comprehensive review of the thread models and attacks against blockchain systems. They classified attacks on blockchain systems into five categories: identity-based attacks, manipulation-based attacks, cryptanalytic attacks, reputation-based attacks, and service-based attacks. Recent blockchain systems are designed with these attacks in mind, and most of the attacks can be defeated if the system is configured following best practices. The design of permissioned consortium blockchains inherently makes many attacks more difficult. For instance, most blockchains use unique transaction IDs and nonces to protect transactions from replay attacks. Permissioned consortium blockchains make Sybil attacks difficult to conduct as identity management is limited to organization administrators.

Regarding DISP, its blockchain security lies within the design and implementation of Hyperledger Fabric. Brotsis et al. [53] highlighted four attack surfaces of Hyperledger Fabric, namely consensus, chaincode, network, and privacy-preserving mechanisms. Hyperledger Fabric is protected against most consensus-oriented attacks but it is more vulnerable to non-deterministic behaviors in chaincode implementation and compromised participants. While the latter type of threat can be eased with careful deployment and maintenance, meticulous attention has been given to the design and implementation of DISP chaincodes to eliminate non-deterministic behavior and ensure the consistency of transactional data. For example, the chaincodes use deterministic JavaScript Object Notation (JSON) serialization libraries to format results. In addition, the application generates all timestamps in the transactions instead of creating them when chaincodes are executing on peers. By doing so, we can eliminate failed transactions due to the system clock not synchronizing across peer nodes. Finally, chaincodes always check the caller’s identity and input parameters to prevent impersonation attacks and invalid requests.

For privacy, the identities and transactions are visible to all consortium participants. Although this is usually expected in a trusted environment, users of DISP have the option to conceal the IoT data with the help of auxiliary storage and encryption. Moreover, the private data collection feature offered by Hyperledger Fabric and zero-knowledge proofs [54] are promising approaches that can improve identity and data privacy in our platform. We leave this as future work.

(2) *Auxiliary storage security and privacy.* We discuss the security and privacy of the auxiliary storage systems using the confidentiality, integrity, and availability (CIA) model. Confidentiality means that the IoT data in the storage should be accessible only to authorized users. In DISP, securely passing sensitive data between a service provider and a consumer can be achieved using a one-time encryption key or access token in terms of data streams. A key may be asymmetrically encrypted using the receiver’s identity and sent via the blockchain. Regarding confidentiality during data transfer, the blockchain and auxiliary storage enforce encryption through Transport Layer Security (TLS). Integrity ensures data authenticity, i.e., no unauthorized alteration to data. The data may be modified accidentally due to system errors or by a malicious party. DISP utilizes blockchain as a layer of data integrity assurance for data in auxiliary storage by virtue of its immutability. Therefore, data providers are encouraged to include a digital digest and signature of the data alongside URI in the service requests and responses. Finally, availability measures how often the data are accessible to its users. For IoT data that desire a high level of availability, distributed storage schemes such as IPFS may be used to facilitate data dissemination and improve data availability. DISP consortium administrators and application developers have the flexibility of realizing data availability.

(3) *IoT device security and privacy.* IoT devices have a long history of being a weak link to IoT system security. Apart from being exposed to physical attacks such as node capturing, sleep deprivation, and false-data injection [55], IoT devices are also vulnerable to network-based attacks. For example, insecurely configured devices are often targeted by IoT botnets [45]. An attacker can acquire access to such devices by brute force attacks or exploiting software flaws. Once successful, malware will be injected into these devices to grow the zombie network or initiate DDoS attacks against other targets.

The proposed DISP can remedy network-based attacks against IoT devices. It offers a secure communication channel to IoT networks that can replace insecure communication protocols such as Telnet and HTTP. The attack surface of IoT devices shrinks as the number of needed services decreases. DISP also eliminates the need for weak credentials by employing strong cryptographic keys and certificates. It also enables the automatic decommission of old IoT devices or decommission of compromised devices using certificate revocation mechanisms. Finally, the use of blockchain and decentralized storage also enhances system security due to the absence of a centralized server, which is often the SPOF in IoT systems.

## 6. Conclusions

This paper presented an innovative platform called DISP for secure and decentralized IoT communications utilizing the consortium blockchain. DISP models IoT communications as services supported by smart contracts. The service provider, usually an IoT device, exchanges messages with service users securely through blockchain transactions. To support a wide range of applications, DISP also incorporates an auxiliary storage system as a secondary communication channel whose data integrity can be assured by the blockchain. Meanwhile, the inclusion of platform SDKs and gateway makes it easy to integrate the proposed platform into existing IoT systems and devices. Furthermore, a prototype implementation as well as exemplary applications are presented to showcase DISP’s generality and versatility. This paper also elaborates on the experimental setup, methodology, and metrics we used to evaluate the performance of DISP. Since the performance of a blockchain system is influenced by a variety of factors, we measure the platform’s transaction throughput, latency, and hardware resource utilization under different blockchain configurations and connection sizes. The results indicate that the performance of read operations primarily depends on message size, while the transaction operation performance is subject to batch size, batch timeout, and connection size. Our proof-of-concept implementation can achieve a throughput of 800 RPS and latency of 50ms for read transactions, and a throughput of 80 TPS and latency of 1s for write transactions when the blockchain parameters are optimized. Overall, our proposed work shows great performance and usability potential as a blockchain-based secure communication platform for IoT.

For future work, one direction will be focused on improving transaction throughput and latency for transaction operations on the platform using state-of-the-art lightweight consensus algorithms. Additionally, we plan to investigate new approaches that integrate auxiliary storage with the blockchain to provide the same level of data security and integrity as blockchain transactions. Finally, we will explore new ideas to address privacy concerns and support private services.

## Figures and Tables

**Table 1:** Comparison of DISP to related research.

Related Work	Domain	Goal	Blockchain	Access Control	Data Storage
[13]	Edge computing	Access control	Hyperledger Fabric	Permissioned	Off-chain
[19]	General IoT	Access control	Hyperledger Fabric	Permissioned	On-chain
[26]	General IoT	Access control	BigchainDB	Permissioned	On-chain
[12]	General IoT	Data management	Hyperledger Fabric	Permissioned	On-chain
[39]	General IoT	Communication	Ethereum	Permissionless	On-chain
[41]	General IoT	Communication	Ethereum	Permissionless	On and off-chain
[11]	General IoT	Communication	Hyperledger Fabric	Permissioned	On-chain
Our work DISP	General IoT	Communication	Hyperledger Fabric	Permissioned	On and off-chain

**Table 2:** Hardware configurations of Hyperledger Fabric nodes and Hyperledger Caliper nodes.

Node Name	CPU (Cores)	Memory (GB)	Disk (GB)	Network (Gbps)	Location
Hyperledger Fabric Orderer1	24	128	233	1	Chicago, IL, USA
Hyperledger Fabric Orderer2	24	128	233	1	Chicago, IL, USA
Hyperledger Fabric Orderer3	24	128	233	1	Chicago, IL, USA
Hyperledger Fabric Org1 Peer1	48	191	447	10	Austin, TX, USA
Hyperledger Fabric Org1 Peer2	48	191	447	10	Austin, TX, USA
Hyperledger Fabric Org2 Peer1	48	191	447	10	Austin, TX, USA
Hyperledger Fabric Org2 Peer2	48	191	447	10	Austin, TX, USA
Hyperledger Caliper Manager	2	2	40	1	Chattanooga, TN, USA
Hyperledger Caliper Worker1	2	2	40	1	Chattanooga, TN, USA
Hyperledger Caliper Worker2	2	2	40	1	Chattanooga, TN, USA
Hyperledger Caliper Worker3	2	2	40	1	Chattanooga, TN, USA
Hyperledger Caliper Worker4	2	2	40	1	Chattanooga, TN, USA
Hyperledger Caliper Worker5	2	2	40	1	Chattanooga, TN, USA

**Table 3:** Performance comparison of the proposed platform to related work.

Related Work	Network Size	Client Size	Read Throughput/Latency	Transaction Throughput/Latency
[13]	5 orderers + 10 peers	3	Not available	Not available/25–183 ms
[19]	Not available	Not available	7–20 RPS/59.5–69 ms	4–19 TPS/161–205.5 ms
[26]	1 BigchainDB server	Not available	Not available	Not available/640–1210 ms
[12]	Not available	50, 150, 250, 500	Not available/271–752 ms	Not available/2490–3012 ms
[39]	Not available	Not available	Not available	Not available
[41]	Not available	Not available	Not available	Not available
[11]	Not available	Not available	2750–3250 RPS/260–600 ms	2250–3250 TPS/300–700 ms
Our work DISP	3 orderers + 4 peers	10, 2000	700–800 RPS/50 ms	18–25 TPS/2000–2500 ms

## Data Availability

Data presented in this study are available at https://github.com/nexus-lab/?q=iot-service-blockchain (accessed on 22 September 2022).

## Abbreviations

The following abbreviations are used in this manuscript:

ABAC	attribute-based access control
ACL	access control list
API	application programming interface
CA	certificate authority
CBAC	capability-based access control
CIA	confidentiality, integrity, and availability
CRL	certificate revocation list
DDoS	distributed denial-of-service
H2M	human-to-machine
ICS	industrial control system
IdM	identity management
IoT	Internet of Things
IPFS	InterPlanetary File System
JSON	JavaScript object notation
M2M	machine-to-machine
MSP	membership service provider
PBFT	practical byzantine fault tolerance
PM	particulate matter
RBAC	role-based access control
RPS	read per second
RTT	round-trip time
SDK	software development kit
SPOF	single point of failure
SUT	system under test
TLS	transport layer security
TPS	transaction per second
URI	UNIFORM RESOURCE IDENTIFIER
UUID	universally unique identifier
